# Behaviors Related to Mosquito-Borne Diseases among Different Ethnic Minority Groups along the China-Laos Border Areas

**DOI:** 10.3390/ijerph14101227

**Published:** 2017-10-15

**Authors:** Chao Wu, Xiaofang Guo, Jun Zhao, Quan Lv, Hongbin Li, Edward B. McNeil, Virasakdi Chongsuvivatwong, Hongning Zhou

**Affiliations:** 1 Yunnan Institute of Parasitic Diseases, Puer 665000, China; wuchao_simao@163.com (C.W); yipdgxf@163.com (X.G.); lvquan2001@163.com (Q.L.); 2 Epidemiology Unit, Faculty of Medicine, Prince of Songkla University, Hat Yai, Songkhla 90110, Thailand; stzhao@163.com (J.Z); edward.m@psu.ac.th (E.B.M.); 3 Hubei University of Medicine, Shiyan 442000, China; 4 Xishuangbanna Prefecture Center of Disease prevention and Control, Jinghong 666100, China; bannacdcjfk@163.com

**Keywords:** behaviors, mosquito-borne diseases, bed nets, repellent, environment, multiple correspondence analysis, ethnic group

## Abstract

*Background*: In China, mosquito-borne diseases are most common in the sub-tropical area of Yunnan province. The objective of this study was to examine behaviors related to mosquito-borne diseases in different ethnic minority groups and different socioeconomic groups of people living in this region. *Methods*: A stratified two-stage cluster sampling technique with probability proportional to size was used in Mengla County, Xishuangbanna Prefecture, Yunnan. Twelve villages were used to recruit adults (≥18 years old) and eight schools were used for children (<18 years old). A questionnaire on behaviors and environment variables related to mosquito-borne diseases was devised. *Results*: Multiple correspondence analysis (MCA) grouped 20 behaviors into three domains, namely, environmental condition, bed net use behaviors, and repellent use behaviors, respectively. The Han ethnicity had the lowest odds of rearing pigs, their odds being significantly lower than those of Yi and Yao. For bed net use, Dai and other ethnic minority groups were less likely to use bed nets compared to Yi and Yao. The odds of repellent use in the Han ethnicity was lower than in Yi, but higher than in Dai. The Dai group was the most likely ethnicity to use repellents. Farmers were at a higher risk for pig rearing and not using repellents. Education of less than primary school held the lowest odds of pig rearing. Those with low income were at a higher risk for not using bed nets and repellent except in pig rearing. Those with a small family size were at a lower risk for pig rearing. *Conclusion*: Different ethnic and socioeconomic groups in the study areas require different specific emphases for the prevention of mosquito-borne diseases.

## 1. Introduction

Yunnan Province, China, located at the southwest border of mainland China with an area of 394,000 km^2^, shares international borders with Myanmar, Vietnam, and Laos. More than 10 types of arboviruses have been isolated in the area and its neighboring Southeast Asia countries. These include Japanese encephalitis virus (JEV), dengue virus (DENV), chikungunya virus (CHIKV), and Zika virus (ZIKV). From 2008 to 2016, DENV outbreaks were reported in Yunnan Province and Laos. Two serotypes (DENV-2 and DENV-3) and CHIKV as well as their co-infections were observed [1]. DENV, causing the 2013 autochthonous outbreak, was shown to be genetically linked to DENV circulating in the north of Laos [2].

Among mosquito-borne diseases (MBD), prevention and control behaviors such as repellent use and bed net use are key actions that individuals can take. The Chinese government has various programs to improve behaviors to reduce the prevalence of mosquito-borne disease in the population. There are 26 ethnic groups settled in China-Laos border areas. In Mengla County (study site), the population of ethnic groups represents 70% of the total. Among them, Dai, Aini, Yao, and Yi are the primary groups. The Fifth Round China of Global Fund Malaria Project in 2007 showed that in Yunnan MBD endemic areas, 65.8% (25,797/39,234) of households owned at least one net and 8.7% (3404/39,234) owned at least one impregnated treated net (ITN) [3]. In 2010, the coverage and use of impregnated treated nets and long-lasting impregnated nets (LLINs) increased to 89.7%, and 30.6% (789/2582) slept under LLINs or ITNs at night [4]. However, the report focused on only three ethnic groups [5,6,7]. There has been no study on how the local residents of different ethnic groups and different socioeconomic groups vary their MBD prevention behaviors. 

In 2016, a survey was conducted to estimate the sero-prevalence of various mosquito-borne diseases in this area. The results on serology are still not available as they need to be verified by highly accurate but time-consuming neutralization tests, in order to minimize the problem of cross-reaction and co-infection. In this communication, we report only the behaviors related to mosquito-borne diseases. As different ethnic groups have different lifestyles [5,6,7], we hypothesize that they will also have different behaviors related to mosquito-borne diseases. The objective of this study was to examine behaviors related to mosquito-borne diseases in different ethnic minority groups and different socioeconomic groups of people living in this region. The information from this study would be used for various mosquito-borne disease prevention and control efforts in this area.

## 2. Materials and Methods 

### 2.1. Ethical Consideration 

Ethical approval was obtained from the Institution Ethical Review Committee of Prince of Songkla University on 2 November 2016 (project code REC 59-244-18-5) and that of the Yunnan Institute of Parasitic Diseases. Informed consent was obtained from all subjects and related authorities including the guardians of subjects under 16 years of age.

### 2.2. Study Site and Study Design

**Figure 1:**
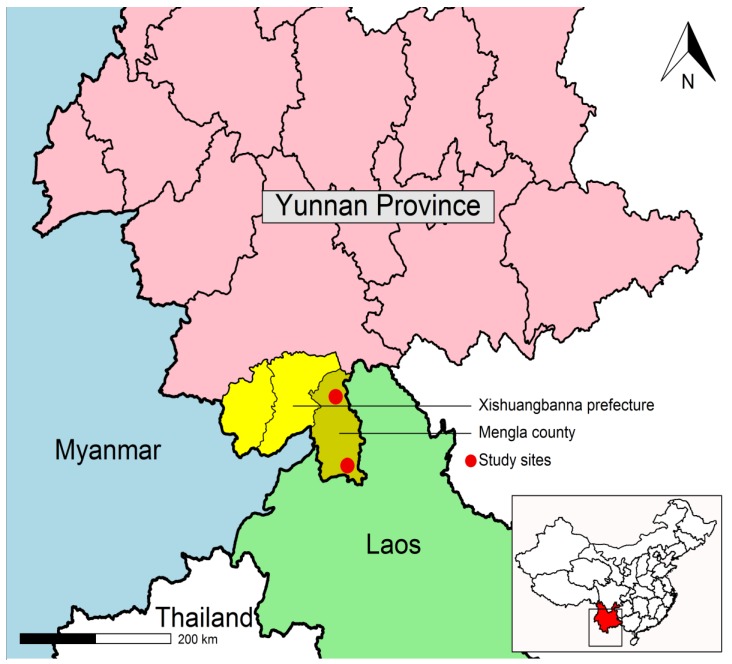
Map of study sites.

The study was conducted in Mengla County of Xishuangbanna Prefecture, Yunnan Province, which has a 678 km border with Laos and a 25 km border with Myanmar as shown in Figure 1. The mean altitude is 1000 m; the annual mean temperature is 21 °C, and the annual total precipitation is 1540 mm. 

A cross-sectional survey was conducted from 12 to 30 September 2016. A structured questionnaire was devised and piloted before being used to collect information. It included the sociodemographic background of the individual behavioral and environmental factors related to mosquito-borne diseases.

### 2.3. Sampling Technique 

A stratified two-stage cluster sampling technique with probability proportional to size was used. The first stratum was adults (≥18 years old). The second was children (<18 years old). In the first stage, five towns of Mengla County were selected randomly using computer software. In the second stage, in each selected town, villages and classes of primary and middle schools were chosen randomly. Finally, 20 clusters were used, which included 12 villages for the adult study and eight schools for the child study.

### 2.4. Study Participants

In each cluster, participants were randomly (using computer software) recruited from the list of adults in each village or a list of 6–18-year-old students in the selected schools. Inclusion criterion was being a resident of the study village for more than six months. Those who had serious hematologic system or immune diseases were excluded.

### 2.5. Questionnaire and Measures

A structured questionnaire was developed to obtain the information from each participant, and was piloted in a village and a school not included in the main study before being used in the field. We developed the same questionnaire for adults and children for the reasons of comparability and possible data pooling in the analysis. The questionnaire contained four sections: (1) sociodemographic characteristics such as age, gender, ethnicity, education level, occupation, family size, and annual family income; (2) environmental variables such as pig rearing by family, distance from house to pig farm, with pig farm near the house, distance of the nearest pig farm, with paddy field, near to the forest, with rubber planting, with discarded tires, with aquatic plants, with pickle jars, with running water, and with tanks for water storage; (3) behavioral bed net use including possessing a bed net, frequency of bed net, and sleeping in a bed net in the daytime; (4) behavioral insect repellent use including using mosquito coil, floral water, and DEET (diethyltoluamide) when working or engaging in activities outside the house.

### 2.6. Data Management and Analysis 

Data were recorded using EpiData (version 3.1, EpiData Association, Odense, Denmark, http://www.epidata.dk). All analysis was performed using R (version 3.4.0, R Foundation for Statistical Computing, Vienna, Austria. https://www.R-project.org).

Twenty behavior variables in the current study were mostly categorical. Multiple correspondence analysis (MCA) was used to reduce the number of these variables into three dimensions using the “FactoMineR” package in R [8,9]. Details of the methods and results are described elsewhere (paper submitted to the Journal of Thai Interdisciplinary Research). The scores of each dimension were extracted and dichotomized as below the factor mean (low score for that dimension) and high score.

Univariate associations between sociodemographic variables and each dimension were explored using tabulation followed by chi-squared test. This was followed by multiple logistic regression analyses to adjust for confounders. The level of association was expressed as an odds ratio, and *p* values below 0.05 were considered as reaching statistical significance.

### 2.7. Sample Size Calculation

To estimate the prevalence of a risk behavior, we assumed 50% prevalence. With 95% confidence interval of the prevalence deviating 6% from the estimate and a design effect of 2, the sample size required for each age group was 534. We initially planned for 10 clusters for each age group, or around 54 participants per cluster. 

## 3. Results

### 3.1. Descriptive Statistics

The actual sample size slightly deviated from what was planned. A total of 1295 participants were included the study, coming from five towns and 20 clusters (eight schools contributing 720 students and 12 villages contributing 545 adults). The personal backgrounds of these two groups are summarized in Table 1. Imbalances in the distribution of gender, ethnic group, family size, and family per capita income of the respondents were seen. We did not test their statistical significance, as these are all settings rather than only those that affect our hypothesis. However, we kept those independent variables for the adjustment of the final results on behavior and environment.

The right part of Table 2 shows the dimension of behavior and environment determined by Multiple Corresponding Analysis using the combined data. The scores in each of the three columns denote the level of contribution of each variable to the dimension. A positive score denotes the same direction of the variable and the dimension. A negative score denotes the opposite direction. Omitted cells with “_” denote very low (<0.025) contribution of the dimension for that variable. 

From the table, variables related to pig farms make a strong positive contribution to Dimension 1. We thereafter considered Dimension 1 as the “Pig rearing” dimension. 

Variables related to bed nets, such as possessing bed nets, using bed nets in general, and using bed nets in the day contribute to Dimension 2. We named this dimension “Bed net use”. It should, however, be noted that sleeping under a bed net during the daytime had an opposite direction compared to possessing bed nets and using bed nets in general.

Using repellents outside the house, using mosquito coils, and using floral water contribute to Dimension 3. We named this dimension “Repellent use”.

It can be noticed that Dimension 3 is also affected to a small extent by the pig rearing variables in a negative direction.

### 3.2. Univariate Analysis among the Sociodemographic Factors and Three Dimensions

To simplify the analysis and for the purpose of effective communication, all three dimensions of behaviors were dichotomized. Pig rearing dimension scores of less than 0 were labeled as “good”, or “bad” if otherwise. The opposite was true for the bed net and repellent use dimensions.

Table 3 summarizes the association between sociodemographic characteristics and each of three dimensions for all subjects. The *p* value for each variable was based on Pearson’s chi squared test for non-ordinal variables such as gender and ethnic group, and on chi-squared test for ordinal variables such as income, family size, and education level.

Gender was not associated with MBD behaviors in any dimension. In contrast, age group was associated with MBD behaviors in all dimensions. Compared with the adult group, the child group was more likely to have pig rearing environment, and not use repellent, but also more likely to use a bed net. 

Compared to Han, the minority groups of Dai, Yi, and Yao were more likely have exposure to a pig rearing environment; Dai and “Others” were more likely to use a bed net, but the Yi ethnic group was less likely to use a bed net, and Aini, Yi, and Yao groups were more likely to use repellent.

Compared to farmer, students and those with other occupations were less likely to be exposed to a pig rearing environment and less likely to use repellent. Students were less likely to use bed nets and “others” more likely to use bed nets.

High education level was associated with low exposure to pig rearing, and higher exposure to bed net use.

Higher annual family income per capita was negatively associated with pig rearing and positively associated with bed net and repellent use. A person from a large family was more likely to rear pigs and less likely to use bed nets. There was no significant relationship between family size and repellent use.

### 3.3. Results from Logistic Regression

Table 4 summarizes the results of logistic regressions predicting “bad” behaviors of each of the three models related to mosquito-borne diseases.

Neither gender nor age group was significantly related to rearing pigs, bed net or repellent use. 

Han and “Other” ethnicities had the lowest odds of exposure to rearing pigs. Their odds were significantly lower than Yi and Yao. For bed net use, Dai and “Other” ethnicities were less likely to use bed nets, in contrast with Yi and Yao. The odds of repellent use behaviors in the Han ethnicity was lower than Yi, but higher than Dai.

Farmers (the reference group) were at higher risk for pig rearing and repellent non-use.

Education of less than primary school held the lowest odds of pig rearing. 

Subjects with low income (≤RMB 8000) were at higher risk for not using bed nets or repellent. 

Subjects with small family size were less likely to rear pigs.

## 4. Discussion

In this study, over half of the subjects were children. Although their ethnic backgrounds were different from those of the adult sample, their MBD-related behaviors were similar. Yi and Aini groups dominated in the adult sample, whereas “Other”, Yao, and Yi contributed significantly to the child group. MCA helped to reduce the number of behavior variables to only three domains, namely, pig rearing environment, bed net use behaviors, and repellent use behaviors. Han and “Other” ethnic groups had the lowest odds of rearing pigs; Yi and Yao were mostly likely to use bed nets and Dai was most likely to use repellent. Farmer was the most likely occupation to rear pigs and not use repellent. Income had no significant relation to pig rearing and bed net use. In general, the middle-income group (CNY¥ 8000–12,000) was more likely to use repellent. 

Pig rearing is a known risk factor for Japanese Encephalitis virus, because pigs are an important amplifying host. Studies conducted in India [10,11,12], Republic of Korea [13,14], Nepal [15], and China [16] showed the same results. The need for the vaccination of local residents and pigs against JEV is often considered [17,18]. However, this is not practical in the study areas. Farmers were more likely to rear pigs because of economic and food needs. Han and “Other” ethnic groups were less likely to rear pigs. The reasons for this ethnic influence and whether these two ethnic groups have lower incidence of JEV is not known.

Bed net use can prevent various mosquito-borne diseases. The most successful field is malaria prevention and control [19,20]. Local health systems have been trying to promote bed net use, especially long-lasting insect treated bed nets for malaria prevention. This was not very successful as the percentage of bed net use was only slightly higher than 50%. Research performed on the use of bed nets among minority groups indicated that human, socioeconomic, and environmental factors can all affect the use of bed nets in China [5,7] and Solomon Islands [6]. Bed net use became compulsory for US soldiers in the Pacific during World War II following severe outbreaks of malaria and dengue fever [21,22]. International health groups are providing long-lasting, insecticide-treated nets to residents in malaria endemic areas of underdeveloped countries, particularly in Africa. In such areas, the regular use of insecticide-treated bed nets can reduce childhood mortality up to 20% and severe disease up to 50% [23,24]. In sub-Saharan Africa, insecticide-treated nets are popular method of malaria control [25]. Our study showed that the relatively high percentage of bed net use among Yi and Yao should be a good example for other ethnic groups to follow.

Repellent use in the study varied considerably. Repellents are used by individuals to reduce the number of bites from hematophagous arthropods [26]. Such products include topical repellents applied directly to the skin, but they also include compounds on clothing, insecticide-treated bed nets, and various devices that emit vapor or droplets into a small space (e.g., mosquito coils) [27]. A study conducted in Yunnan, China, revealed that personal protection is widely used and accepted, with the major barrier to its use being affordability [28]. Research conducted in India showed that repellents are widely used in India. Their using is influenced by the level of education and socioeconomic status [29]. The current study indicated that the Dai ethnicity was more likely to use repellent, probably because they spend a lot of time in rubber plantations.

## 5. Limitation

This study was based on a questionnaire, not direct observation. More than half of the respondents were children, who might give inaccurate information. The extent of information bias is unknown. Thus, this cross-sectional survey may capture only a snapshot of information about the participants; the findings may change with time.

## 6. Conclusions

Along China-Laos border areas, ethnic minority groups vary their exposure to pig rearing, their bed net use behaviors, and repellent use behaviors. The behaviors are also influenced by other sociodemographic factors. These influences should be taken into account in the control of mosquito-borne diseases.

## Figures and Tables

**Table 1:** Sociodemographic characteristics.

Characteristic	Adult Group (>18 Years)		Child Group (≤18 Years)	
	Frequency	Percent	Frequency	Percent
Number of clusters	12	60.0	8	40.0
Number of participants	565	43.6	730	56.4
Gender				
Male	185	32.7	397	54.4
Female	380	67.3	333	45.6
Education level				
Less than primary school	214	37.9	14	1.9
Primary school	310	23.0	405	55.5
Secondary school or above	221	39.1	311	42.6
Ethnicity				
Han	55	9.7	93	12.7
Dai	105	18.6	82	11.2
Aini	125	22.1	102	14.0
Yi	157	27.8	144	19.7
Yao	102	18.1	154	21.1
Other	21	3.7	155	21.2
Occupation				
Farmer	453	80.2	8	1.1
Student or other	112	19.8	722	98.9
Family size				
≤3	127	22.5	70	9.6
4–5	258	45.7	441	60.4
≥6	180	31.9	219	30.0
Annually family income (CNY¥ per capita)				
≤8000	193	34.2	123	16.8
8000–12,000	152	26.9	254	34.8
12,000–15,000	118	20.9	249	34.1
≥15,000	102	18.1	104	14.2

**Table 2:** Comparison of environmental factors and personal behaviors between the child group and adult group.

Variables	Child Group (Age ≤ 18 Years)	Adult Group (Age > 18 Years)	Dimension 1	Dimension 2	Dimension 3
	%	%	Contribution Score		
Pig rearing by family					
Yes	60	61.2	0.574	_	−0.379
No	40	39.8	−0.88	_	0.582
Distance from house to pig farm					
Side of the house	54.2	57.7	0.561	_	−0.413
Less than 3 km	5.8	3.4	0.97	_	_
Without pig rearing	40.0	38.9	−0.876	_	0.584
With pig farm near the house					
Yes	34.9	42.5	0.921	_	−0.349
No	65.1	57.5	−0.57	_	_
Distance of the nearest pig farm					
Side of the house	28.1	34.2	1.022	_	−0.425
Less than 3 km	6.8	8.3	0.722	−0.939	_
Without pig rearing	65.1	57.5	−0.57	_	_
Housing structure					
Wooden/bamboo structure	24.2	19.6	_	_	_
Brick and cement structure	71.4	77.5	_	_	_
Other	4.4	2.8	_	_	_
With paddy field					
Yes	69.9	81.8	_	_	_
No	30.1	18.2	_	_	_
Near to the forest					
Yes	23.2	25.7	_	_	_
No	76.8	74.3	_	_	_
With rubber planting					
Yes	60.4	68.1	_	_	_
No	39.6	31.9	_	−0.47	_
With discarded tires					
Yes	17.1	20.0	_	_	_
No	82.9	80.0	_	_	_
With aquatic plants					
Yes	17.5	14.0	_	_	_
No	82.5	86.0	_	_	_
With pickle jars					
Yes	75.8	82.3	_	_	_
No	24.2	17.7	_	_	_
Running water					
Yes	92.3	98.4	_	_	_
No	7.7	1.6	_	_	_
Tanks for water storage					
Yes	36.4	49.4	_	_	_
No	63.6	50.6	_	_	_
Family possesses bed nets					
Yes	70.4	58.9	_	0.558	_
No	29.6	41.1	_	−1.504	_
Often using bed nets					
Yes	55.3	29.7	_	0.921	_
No	44.7	70.3	_	−0.729	_
Sleeping in bed net during daytime					
Yes	64.8	81.4	_	−0.548	_
No	35.2	18.6	_	1.18	_
Using insect repellent when working outsides					
Yes	48.2	50.1	0.603	_	0.739
No	51.8	49.9	−0.58	_	−0.711
Using mosquito coils					
Yes	54.8	54.2	0.63	_	0.753
No	45.2	45.8	−0.526	_	−0.628
Using florial water when working/playing outside					
Yes	72.9	80.0	0.711	_	1.071
No	27.1	20.0	_	_	−0.339
Using DEET when working/playing outside					
Yes	1.5	4.6	_	_	_
No	98.5	95.4	_	_	_

Note: “_” variables are those which contributed less than 2.5% to three dimensions. The negative contribution scores denote the opposite direction.

**Table 3:** Univariate analysis of association between the sociodemographic factors and pig rearing, bed net use, and repellent use.

Socio-Demographic Factors	Pig Rearing			Bed Net Use			Repellent Use		
	Bad/Good	OR 95% CI	*p*-Value	Bad/Good	OR 95% CI	*p*-Value	Bad/Good	OR 95% CI	*p*-Value
Gender									
Male	262/320	1	0.078	305/277	1	0.091	305/277	1	0.502
Female	356/357	1.22 (0.97, 1.53)		340/373	0.83 (0.66, 1.04)		387/326	1.08 (0.86, 1.35)	
Age group									
Adult	289/276	1	0.03	250/315	1	<0.01	377/188	1	<0.01
Child	329/401	1.28 (1.02, 1.6)		395/335	0.67 (0.54, 0.84)		315/415	2.64 (2.09, 3.34)	
Ethnicity									
Han	56/92	1	<0.01	69/79	1	<0.01	69/79	1	<0.01
Dai	98/89	1.81 (1.14, 2.88)		130/57	2.6 (1.63, 4.19)		70/117	0.69 (0.43, 1.09)	
Aini	108/119	1.49 (0.96, 2.33)		127/100	1.45 (0.94, 2.25)		140/87	1.84 (1.19, 2.86)	
Yi	151/150	1.65 (1.09, 2.53)		100/201	0.57 (0.37, 0.87)		193/108	2.04 (1.34, 3.11)	
Yao	151/105	2.36 (1.53, 3.66)		93/163	0.65 (0.42, 1.01)		149/107	1.59 (1.04, 2.45)	
Other	54/122	0.73 (0.45, 1.18)		126/50	2.88 (1.78, 4.69)		71/105	0.77 (0.49, 1.23)	
Occupation									
Farmer	272/189	1	<0.01	221/240	1	<0.01	333/128	1	<0.01
Student	327/398	0.57 (0.45, 0.73)		393/332	1.29 (1.01, 1.64)		310/415	0.29 (0.22, 0.37)	
Other	19/90	0.15 (0.08, 0.25)		31/78	0.43(0.26, 0.69)		49/60	0.31 (0.2, 0.49)	
Education level			0.146 *			0.7846 *			<0.01 *
Less than primary school	120/108	1	0.252	113/115	1	0.942	155/73	1	<0.01
Primary school	252/283	0.8 (0.58, 1.11)		264/271	0.99 (0.72, 1.37)		269/266	0.48 (0.34, 0.67)	
Secondary school or above	246/286	0.77 (0.56, 1.07)		268/264	1.03 (0.75, 1.43)		268/264	0.47 (0.34, 0.67)	
Annual family income per capita (RMB)			<0.01 *			0.017 *			<0.01 *
≤8000	174/142	1	<0.01	168/148	1	0.015	209/107	1	<0.01
8000–12,000	209/197	0.87 (0.64, 1.18)		204/202	0.89 (0.66, 1.21)		194/212	0.47 (0.34, 0.64)	
12,000–15,000	168/199	0.69 (0.5, 0.94)		191/176	0.96 (0.7, 1.31)		172/195	0.45 (0.33, 0.62)	
≥15,000	67/139	0.39 (0.27, 0.58)		82/124	0.58 (0.4, 0.84)		117/89	0.67 (0.46, 0.98)	
Family size			<0.01 *			<0.01 *			0.736 *
≤3	67/130	1	<0.01	75/122	1	<0.01	105/92	1	0.863
4–5	320/379	1.64 (1.16, 2.32)		343/356	1.57 (1.12, 2.2)		378/321	1.03 (0.74, 1.43)	
6	231/168	2.66 (1.84, 3.87)		227/172	2.14 (1.49, 3.09)		209/190	0.96 (0.67, 1.38)	

* Chi-squared test for trend in proportion. OR: odds ratio. CI: confidence interval.

**Table 4:** Associations between sociodemographic factors and bad MBD behaviors.

Predictive Factors	Model 1 Pig Rearing	Model 2 Not Using Bed Net	Model 3 Not Using Repellent
	AOR 95% CI	AOR 95% CI	AOR 95% CI
Age group: Adult vs Child	1.23 (0.4, 3.76)	1.04 (0.35, 3.07)	0.78 (0.25, 2.44)
Gender: Female vs Male	1.16 (0.91, 1.48)	0.87 (0.68, 1.11)	0.84 (0.66, 1.07)
Ethnicity ref.: Han	***	***	***
Dai	1.41 (0.88, 2.27)	2.47 (1.53, 3.97) **	0.45 (0.28, 0.74) **
Aini	1.14 (0.73, 1.78)	1.32 (0.85, 2.03)	1.32 (0.84, 2.06)
Yi	1.58 (1.02, 2.42) *	0.55 (0.36, 0.84) **	1.59 (1.04, 2.43) *
Yao	1.86 (1.19, 2.9) **	0.52 (0.34, 0.8) **	1.25 (0.8, 1.94)
Other	0.64 (0.4, 1.04)	2.44 (1.52, 3.92) **	0.9 (0.57, 1.43)
Occupation Reference: Farmer	***	***	***
Student	0.67 (0.22, 2.07)	1.32 (0.44, 3.93)	0.21 (0.07, 0.68) **
Other	0.15 (0.08, 0.27) **	0.39 (0.22, 0.67) **	0.28 (0.17, 0.48) **
Education level Reference: Less than primary school	***		
Primary school	1.5 (1.01, 2.22) *	0.85 (0.57, 1.26)	1.02 (0.66, 1.55)
Secondary school or above	1.99 (1.33, 2.96) **	1.06 (0.71, 1.59)	1.04 (0.68, 1.6)
Annual family income (per capita RMB) ref.: ≤8000 (RMB)			***
8000–12,000	0.98 (0.71, 1.35)	0.67 (0.48, 0.93) *	0.61 (0.44, 0.85) **
12,000–15,000	0.9 (0.64, 1.26)	0.77 (0.55, 1.08)	0.66 (0.46, 0.93) *
>15,000	0.67 (0.43, 1.04)	0.68 (0.44, 1.05)	1.04 (0.67, 1.62)
Family size ref. ≤3	***		
4–5	1.41 (0.98, 2.04)	1.17 (0.81, 1.68)	1.33 (0.92, 1.92)
≥6	2.11 (1.4, 3.18) **	1.4 (0.93, 2.11)	1.34 (0.88, 2.02)

AOR: Adjusted odds ratio; 95% CI: confidence interval; * *p* (Wald’s test) < 0.05; ** *p* (Wald’s test) < 0.01; *** *p* (LR-test) < 0.05. Model 1: Associations between sociodemographic factors and environmental pig rearing; Model 2: Associations between sociodemographic factors and bed net use behaviors; Model 3: Associations between sociodemographic factors and repellent use behaviors.

## Abbreviations

MBD	mosquito-borne diseases
MCA	multiple correspondence analysis
